# Intensive group treatment for children with selective mutism: a pilot study with repeated assessments

**DOI:** 10.1186/s13034-026-01203-4

**Published:** 2026-10-27

**Authors:** Christina Schwenck, Megan Williams, Jana Zimmermann-Hauel, Carolin Röse-Werkmann, Gloria Gehb, Naomi L. Werkmann

**Affiliations:** Department of Clinical Child and Adolescent Psychology, Justus-Liebig-University Giessen, Otto-Behaghel-Str. 10, 35394 Giessen, Germany

**Keywords:** Selective mutism, Intensive treatment, Group treatment, Child anxiety

## Abstract

**Background:**

Selective mutism (SM) is an anxiety disorder characterized by a persistent failure to speak in specific social situations despite speaking normally in others. Preliminary evidence suggests that intensive behavioral group interventions may be a promising treatment approach. This pilot study evaluated the outcomes of such an intervention for children with SM for the first time in a German clinical sample.

**Methods:**

Sixteen children aged five to nine with SM were included in the study and fifteen participated in an intensive group intervention based on Parent-Child-Interaction Therapy for Selective Mutism (PCIT-SM). Two baseline, one post-treatment, and one three-month follow-up assessments were conducted. Primary outcome was parent-rated SM symptom severity assessed with the Frankfurt Scale for Selective Mutism (FSSM). Remission status based on a structured clinical interview served as secondary outcome. Changes over time were analyzed using linear mixed-effects models and Cochran’s Q tests.

**Results:**

SM symptom severity decreased significantly over time with large effects from baseline 1 to post-treatment (*d*_*RM*_ = 1.03) and follow-up (*d*_*RM*_ = 1.69). No significant differences emerged between the two baseline assessments. Remission rates increased from 0% at first baseline to 47% at follow-up. A significant overall change in diagnostic status across assessments was observed. Exploratory analyses further showed that general anxiety decreased from baseline to follow-up.

**Conclusions:**

The findings provide preliminary support for intensive group interventions based on PCIT-SM principles as a promising treatment for children with SM. Future controlled studies with larger samples and longer follow-up periods are needed to evaluate treatment mechanisms and long-term outcomes.

*Trial registration* German Clinical Trials Register (DRKS), DRKS00040917, retrospectively registered on 13 July 2026.

## Plain language summary

Children with selective mutism (SM) often speak normally at home but remain silent in situations such as school or in interactions with unfamiliar people. In this study, children participated in an intensive group treatment program that helped them practice speaking in social situations with support of therapists and parents. Symptoms of SM improved over time, and nearly half of the children no longer met diagnostic criteria for SM three months after treatment. These findings suggest that intensive group treatment may be a promising approach for children with SM.

## Introduction

Selective mutism (SM) is an anxiety disorder characterized by the persistent failure to speak in certain social situations like at school or with strangers. At the same time speaking behavior is unaffected in other social settings like at home or with confidants [1]. The mean age of onset lies in early childhood, and symptomatology is associated with significant impairment in social, educational and later occupational functioning [2, 3]. Longitudinal studies further indicate frequent negative long-term outcomes, specifically if SM is left untreated [2, 4]. In addition, SM is often accompanied by other anxiety disorders, particularly social anxiety disorder [5].

Despite its clinical relevance and an estimated prevalence between 0.18 and 1.90% [3], SM is highly understudied and underdiagnosed. Consequently, both the evidence base regarding effective interventions and the understanding of significant targets for treatment are still limited.

### Potential underlying mechanisms of failure to speak as targets for intervention

Despite the scarce literature on underlying processes in SM, avoidance has frequently been discussed as a potential contributor to the failure to speak in social situations. Avoidance is consistently found in anxiety disorders in general and in social anxiety disorder in particular [6]. Preliminary evidence suggesting that avoidance also plays a significant role in SM has been found in both qualitative and quantitative studies [7, 8]. In behavioral therapy, avoidance is typically addressed through exposure-based interventions, in which gradual approaches toward a desired target behavior are positively reinforced. Newly acquired behaviors are then generalized through repeated practice across different situational contexts [9]. In line with this, a network analysis of SM symptoms identified silence and its situational selectivity as two of the three most central symptoms, suggesting that they may be particularly relevant for the maintenance of SM symptomatology. Therefore, practicing verbal communication in different social contexts could be an important treatment target to improve overall symptomatology [10].

A second possible mechanism underlying the failure to speak is an altered physiological stress response in speech demanding situations associated with the inability to engage in active coping mechanisms towards perceived threat. In line with this, children with SM have frequently been described as frozen in speech demanding situations in qualitative and questionnaire studies [7, 10, 11]. Notably, the network analysis by [12] identified motor inhibition as the third central symptom, highlighting the potential of both avoidance and behavioral freezing in the maintenance of SM symptomatology. Furthermore, a study adopting an experimental design and assessing peripheral physiological markers found altered arousal patterns in children with SM [8, 12, 13]. In such a state of behavioral freezing, it may not be possible for patients to immediately engage in exposure therapy. Children may first need support in overcoming this initial inhibition before they can effectively engage in verbal exposure exercises.

In summary, two possible mechanisms that may contribute to silence can be identified: avoidance and behavioral freezing. Importantly, these mechanisms may call for different therapeutic approaches. Whereas avoidance-related processes are typically addressed through direct exposure to speech-demanding situations, pronounced behavioral freezing may initially interfere with a child’s ability to engage in exposure exercises effectively and may therefore require prior reduction of stress. This might make treatment particularly challenging, given the possibility that both processes could occur simultaneously and potentially interact with or reinforce one another.

### Behavioral interventions targeting silence in SM

Studies examining the effects of psychotherapy for SM are generally scarce, and almost all of them focus on (cognitive-)behavioral therapy interventions. A meta-analysis on *k* = 5 randomized controlled trials (RCTs) with *N* = 233 children with SM and treatment durations between one week and six months found a large overall effect size of *g* = 0.87 [95% CI: 0.58, 1.16] [14]. Interventions included in this analysis contained different measures to encourage speech production such as contingency management, exposure, shaping or cognitive measures. However, given the underdiagnosis of SM, recruiting sufficiently large samples for RCTs remains challenging [3]. In addition, delaying treatment in affected children may raise ethical concerns. Consequently, several studies have adopted a repeated-measures design with two baseline assessments as an alternative methodological approach. Such designs may be particularly suitable in SM research because untreated SM symptomatology has been shown to remain relatively stable over time, allowing symptom changes during the intervention phase to be interpreted more confidently as treatment-related effects [2, 15, 16].

Besides (cognitive-) behavioral therapy, one approach that has demonstrated promising results in an individual therapy setting is the parent-child-interaction therapy for SM (PCIT-SM) [17]. Notably, PCIT-SM incorporates techniques aimed at reducing stress as well as exposure-based techniques [17]. A study that evaluated this behavioral intervention with *N* = 31 children over 16 weekly sessions found a large effect on the general speaking behavior of participants (*d* = 1.8 pre-post). This positive effect was also maintained at three-month and one-year follow-up assessments [18].

### Intensive group interventions for children with SM

Intensive group interventions may be particularly suitable for children with SM because they provide repeated opportunities for exposure to speech-demanding social situations and interactions with unfamiliar peers and adults in a naturalistic setting. In addition, the intensity of the format may allow sufficient time for children to gradually reduce behavioral freezing before engaging in exposure. To date, four studies have evaluated PCIT-SM in an intensive group format, three of which use a repeated-measures design with two baseline assessments. Methodological details of the studies are shown in Table 1. Overall, findings have been promising but somewhat heterogeneous. Only one study adopted an RCT between baseline and post-assessment with a waiting-list control group. Although no significant effect on remission rate was found at post-assessment, the authors reported positive effects on clinical global impression (50% responders in the treatment group vs. 0% in the control group, φ = − 0.58). In addition, positive effects were found for parent-rated SM symptomatology in social settings (*d* = 0.58), social anxiety severity (*d* = − 0.50) and global functioning (*d* = 0.73) with medium effect sizes. At follow-up (non-RCT) the remission rate was 46% [19]. Another study assessing a slightly older sample (10–17 years) did not include diagnostic status as an outcome measure but found medium to large effects with respect to parent- and self-reported questionnaire data on SM symptomatology at two and five months post-treatment (parent ratings: Hedges’ *g* = 1.32 and 2.09; adolescent ratings: Hedges’ *g* = 0.59 and 1.14), as well as secondary outcomes [20]. In contrast, a study evaluating a remote intensive group intervention did not find significant improvements in parent-reported SM symptomatology, although 44.4% of participants were rated as treatment responders on a clinician-rated global impression measure [21]. Finally, a fourth study found very good acceptability and treatment integrity for the intensive group treatment. In contrast, speech behavior and anxiety ratings improved only in a subset of the 25 children included, and the overall changes were not significant [22].

### The current study

Taken together, preliminary evidence suggests that behavioral interventions may be effective for children with SM and that intensive group interventions represent a potentially promising treatment approach. PCIT-SM may well be suited to address different processes potentially underlying silence in SM, including avoidance-related processes and altered physiological arousal.

However, the existing literature remains limited by small sample sizes, heterogeneous intervention formats, and inconsistent reporting of clinically meaningful outcomes such as responder status or remission rate based on diagnostic gold standards. In addition, all studies on intensive group-based interventions for SM have been conducted in North America, limiting the generalizability of findings to other healthcare systems and clinical contexts. Evaluating this intervention in a German clinical sample may therefore contribute to determining whether findings generalize beyond North American healthcare settings and support the broader implementation of intensive group treatment for children with SM.

Therefore, the current study aimed to extend the existing literature by evaluating an intensive group intervention for children with SM in a German sample, while incorporating clinically meaningful outcome measures, including remission. In our study, we adopted a repeated-measures design with two baseline assessments, one post-assessment and one follow-up assessment three months after treatment. Based on previous findings, we expect to find improvements in the severity of SM symptomatology (primary outcome) and remission rate (secondary outcome) over time with significant differences between baseline 1 and follow-up assessments with respect to both outcomes. For SM symptomatology we further expect improvements with respect to baseline 1 and post-assessment. The effects on general anxiety and correlations with treatment outcome will be assessed in an exploratory manner.

**Table 1:** Methodological characteristics of studies assessing the outcomes of intensive group treatment for selective mutism [19–21]

	Cornacchio et al. [19]	Hong et al. [21]	Haggerty et al. [22]	Kupferberg et al. [20]
Sample size *N*	29	9	25	16
Duration/frequency	5 days intensive treatment	5 days intensive treatment remote	5 days intensive treatment	5 days intensive treatment
Age range participants in years	5-9	4-10	4 - 11	10-17
Study design	RCT from pre to post with waiting list control group	repeated measures design, no control group	repeated measures design, no control group	repeated measures design, no control group
Dependent variables	clinical interview (remission rate), questionnaires, clinical global impression	clinical interview (remission rate), questionnaires, clinical global impression	questionnaires, behavioural observations	clinical interview (remission rate), questionnaires

## Methods

### Sample

**Fig. 1:**
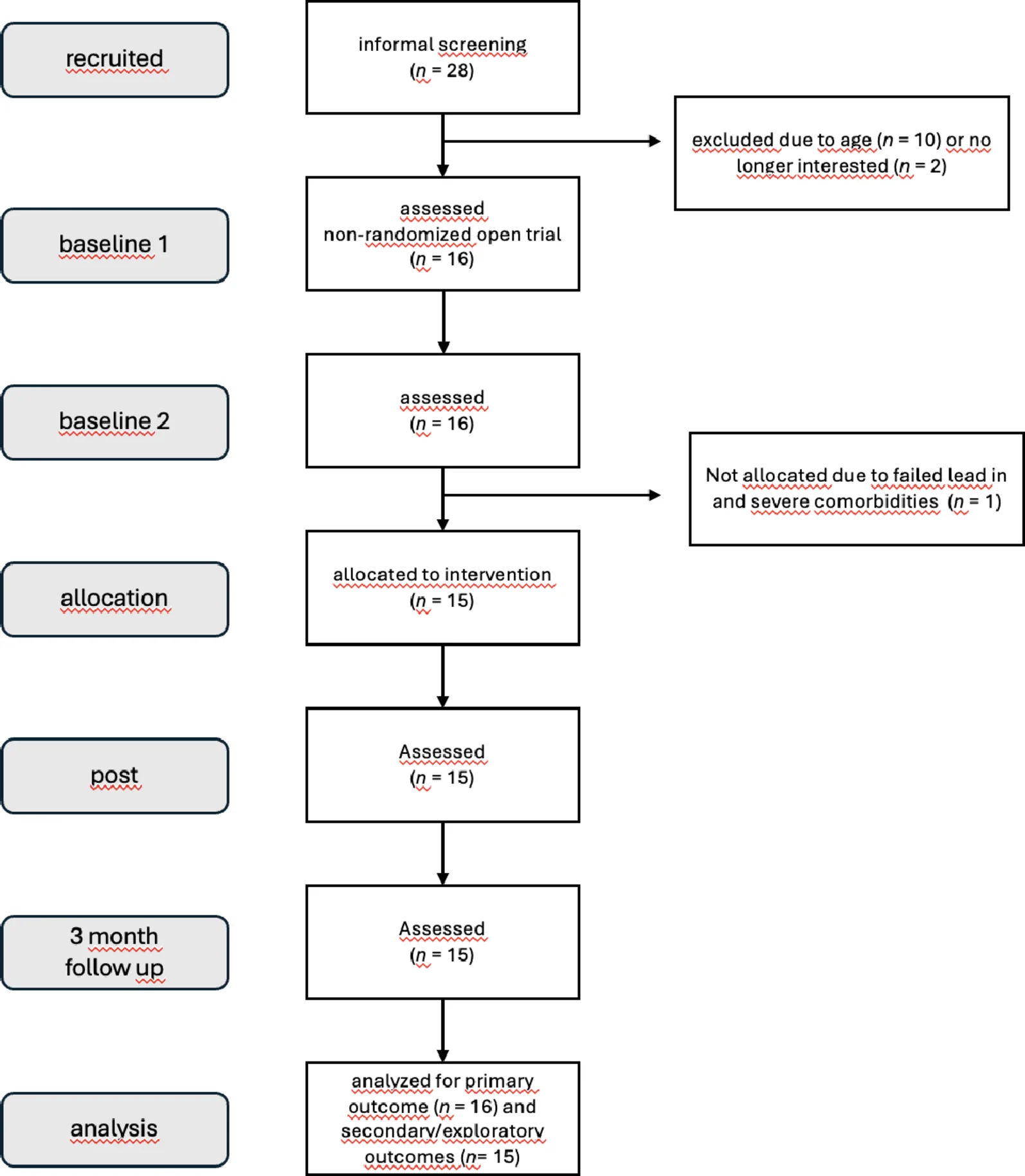
*CONSORT* flow diagram of participant flow

Inclusion criteria comprised a primary diagnosis of SM according to DSM-5, an age of five to ten years, sufficient German language skills of the child and at least one parent, and informed consent. In total, *N* = 28 children were screened for participation, all of them fulfilled inclusion criteria. After screening, two families were no longer interested. Of the remaining 26 families, *n* = 16 children (*n* = 10 female) were included in the study. Participants were selected based on age compatibility to achieve a relatively homogeneous age distribution within each group. Children aged five to nine years (*M* = 7.02, *SD* = 1.24) participated. One child completed both baseline assessments but did not participate in the intervention because it became obvious during the first week that another diagnosis was the primary concern and needed to be treated first. This decision was made jointly with the child’s parents in the best clinical interest of the child and was independent of the child’s SM symptom severity or subsequent study outcomes. A flowchart of participants is presented in Fig. 1.

Five children were bilingual, and three children had received individual psychotherapy. In one case, treatment continued throughout all assessment points, whereas in the other two cases, treatment had been completed before baseline. On average, children had *M* = 1.53 (*SD* = 0.99, range 0–4) comorbid diagnoses other than SM according to a clinical interview at baseline. These diagnoses were social anxiety disorder (*n* = 11), attention-deficit/hyperactivity disorder (*n* = 1), oppositional defiant disorder (*n* = 1), Tic-disorder (*n* = 2), separation anxiety disorder (*n* = 3), specific phobia (*n* = 3), and sleeping disorder (*n* = 2). Psychopharmacotherapy use was not reported by any of the families. Although no standardized socioeconomic status (SES) index was assessed, parental educational attainment and current employment status were collected to characterize the family background. The sample was characterized by a high educational level: 75.0% of mothers and 87.5% of fathers held a university degree. The remaining parents had completed upper secondary education, vocational qualifications, or a secondary school degree; none reported only a lower secondary school qualification or no formal educational qualification. Regarding employment, most fathers were employed full-time (81.3%), whereas most mothers worked part-time (75.0%). One mother reported being a homemaker, and several parents were self-employed or worked as freelancers. No parent reported being unemployed or actively seeking employment. Table 2 presents further sample characteristics at baseline according to two screening questionnaires regarding general psychopathology (CBCL) and SM symptomatology (FSSM; see Assessments).

**Table 2:** Sample characteristics at baseline

		*M* (*SD*)
CBCL (T-values)	Total	57.44 (9.57)
	Internalizing	59.63 (8.50)
	Externalizing	54.06 (10.14)
FSSM	diagnostic scale sum score	8.00 (1.24)
	severity scale home mean score	1.37 (0.60)
	severity scale public mean score	2.36 (0.60)
	severity scale kindergarten/school mean score	2.43 (0.62)

*CBCL* Child behavior checklist, *FSSM * Frankfurt scale for the assessment of selective mutism

### Study procedure

The study followed CONSORT recommendations for pilot and feasibility studies where applicable. The data were collected from two cohorts between 2023 and 2024. The study and intensive group treatment were advertised via social media and support groups, as well as in outpatient clinics for clinical child and adolescent psychology. Interested parents signed up for initial consultation via an online form, during which they were asked about SM symptoms and relevant comorbidities in an unstructured manner and were informed about the study and intervention procedures and requirements. They then had the opportunity to decide whether to participate. Participants were selected based on their age to obtain a sample with a relatively homogeneous age distribution. The study was approved by the local ethics committee, and all final participants provided their informed consent.

### Assessment procedure

Four assessments were conducted: two baseline assessments, a post-treatment assessment, and a three-month follow-up assessment. At the first baseline, the full clinical interview and the questionnaire-based assessment were conducted. At all subsequent assessment time points, the clinical interview was used to reassess all areas of concern identified at the first baseline, and the questionnaire-based assessment was also administered. Parents were encouraged and reminded to fill in the questionnaires within one week; however, the intervals differed slightly between cohorts and individuals with *M* = 35.56 (*SD* = 16.28) days between first and second baseline, *M* = 33.93 (*SD* = 5.96) days between baseline 2 and post assessment, and *M* = 96.87 (*SD* = 12.48) days between post assessment and follow-up.

### Intervention procedure

**Fig. 2:**
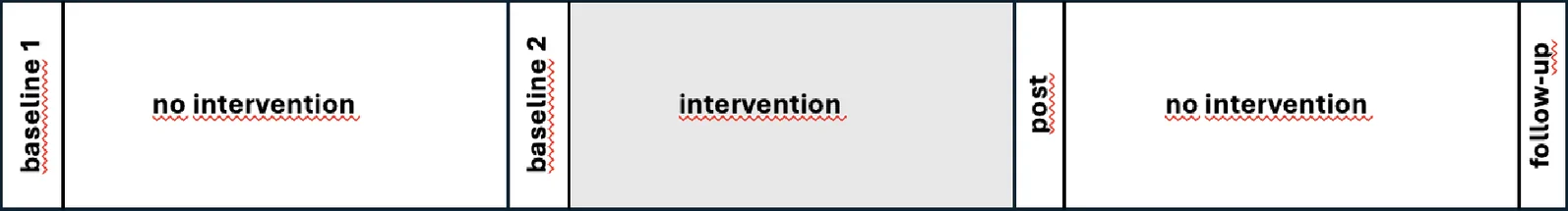
Assessment and intervention procedure

No intervention took place between baseline 1 and baseline 2; between baseline 2 and the post-assessment, parents participated in a parent-training session and children attended the intensive group treatment. Immediately after the group treatment and shortly after the post-measurement, a written report summarizing the diagnostic findings and recommendations for further support was provided, along with an individual feedback session to discuss the results and address any questions. Following the follow-up assessment, an online group meeting was offered to all parents. Figure 2 demonstrates the assessment and intervention procedure.

### Treatment and staff training

PCIT-SM procedures and staff training were closely based on protocols described in other studies [19]. For this reason, the procedure is presented here only in summary; more details regarding the PCIT-SM procedure and staff training can be found in the study by Cornacchio et al. [19]. Although no formal treatment manual was available, a standardized session plan was used consistently across the two cohorts. All counselors received comprehensive training in PCIT-SM by the first author. During the intervention, the team met daily for supervision and case discussion, with the first author present throughout.

#### Parent training

All parents received parent training after baseline 2 comprising an online psychoeducative self-study tool and three online sessions of group-based parent training including breakout groups for practical training in PCIT-SM. During the intensive treatment week, the parents attended a daily one-hour parent training session Monday through Wednesday. On Thursday and for part of Friday, all parents participated in the treatment sessions alongside their children and received live coaching on how to use PCIT-SM to help their children to speak with strangers.

#### Lead-in week

The lead-in week took place before the actual group intervention and the goal of the lead-in week was for the child to speak with two unfamiliar counselors and in front of another child. One parent attended at least two sessions with the child, but families were invited more frequently if necessary to achieve the goal. During the sessions, parents and children engaged in play activities while receiving guidance from a counselor in an adjacent room. Using the PCIT-SM techniques they had learned, the parents helped their children talk to a stranger - the counselor - who gradually approached the play scene. Once the child was able to verbally interact with the counselor, a second session was conducted in which a question was posed in the presence of another child and counselor, with the goal of eliciting a verbal response from the child.

#### Intensive group treatment

Seven children participated in one group, eight in the other. Monday to Friday, children participated in the treatment for six hours daily. Throughout the day, opportunities to speak were created in various social situations, and the children were supported in speaking using PCIT-SM. These situations included, for example, speaking with unfamiliar children, unfamiliar adults, one-on-one, and in front of the group, in public, and with authority figures. Speaking behavior was expanded individually and gradually. Each child had their own counselor for the day, though the counselor changed every day.

#### Counselor team composition

The counselor team comprised one licensed psychotherapist for children and adolescents, who was a certified PCIT-SM trainer. Two psychotherapists in training in the first cohort and three in the second, both with advanced experience in individual psychotherapy, were part of the team of counselors. In addition, nine, respectively ten, psychology students in the clinical master’s program served as counselors; they had previously been trained in PCIT-SM and the treatment of children with SM as part of a university seminar.

### Assessments

#### Frankfurt scale for the assessment of selective mutism (FSSM)

The FSSM [23] is a parent-rated questionnaire developed to diagnose and evaluate SM in children aged between 3 and 18 years. There are different versions for kindergarteners aged 3 to 7 years, schoolchildren aged 6 to 11 years, and teens aged 12 to 18 years. The questionnaire consists of a Diagnostic Scale with ten yes-no questions about the children’s overall speaking behavior. The Severity Scale comprises 41 or 42 questions regarding the specific speaking behavior at kindergarten/school, in public, and at home that are answered on a 5-point Likert scale. An evaluation of the FSSM revealed excellent reliability scores (α = 0.90 − 0.98) and validity with a one-factor solution for the Severity Scale [24]. For the current study, age-appropriate versions were used. The mean sum score of the Severity Scale served as the primary outcome measure.

#### Diagnostic interview for mental disorders in children and adolescents (Kinder-DIPS)

Kinder-DIPS [25] is a structured clinical interview used to diagnose mental disorders in children and adolescents according to both DSM-5 and ICD-10. The interview has a good to very good interrater reliability [26]. In the current study, the Kinder-DIPS was used to assess SM and comorbidities at baseline and SM as clinical outcome.

#### The Bochum anxiety assessment for preschool and elementary school-aged children (BAV 3–11)

The BAV 3–11 [27] is a standardized measure of childhood anxiety for children aged 3 to 11 years. It assesses fears across multiple domains, anxiety-related coping strategies, and physiological symptoms associated with anxiety. The instrument consists of a structured child interview and a parent-report questionnaire. Initial validation studies reported acceptable to good internal consistencies (α = 0.64–0.86), a theoretically meaningful factor structure, and evidence for criterion validity through associations with established measures of child anxiety and parent ratings [27, 28]. In the current study, the parent-report questionnaire was used as an outcome measure, the child interview was not conducted.

#### Child behavior checklist (CBCL)

The CBCL [29] is a parent-report measure for screening of mental health related symptomatology in children and contains 113 items that must be rated on a three-point Likert scale. In the present study, the parent-report version for preschool children (CBCL 1.5-5R) as well as for school children (CBCL 6-18R) were employed. The CBCL has shown excellent psychometric properties, including high internal consistencies for the broadband scales Internalizing Problems (α ≈ 0.90), Externalizing Problems (α ≈ 0.94), and Total Problems (α ≈ 0.97), as well as strong evidence for construct and criterion validity [30]. The German adaptation demonstrated similarly satisfactory psychometric properties and provides age- and gender-specific norms for German-speaking populations [29]. For the present study, the broadband scales Internalizing Problems and Externalizing Problems, as well as the Total Problems score, were used to characterize the sample.

#### Retrospective infant behavioral inhibition scale (RIBI)

The RIBI [31] is a parent-report questionnaire that assesses a child’s behavioral inhibition during the first two years of life retrospectively. It includes 20 items that are rated on a 5-point Likert scale. The scale has excellent reliability (α > 0.90) and convergent validity has been demonstrated [31].

### Statistical analyses

Changes in SM symptom severity (primary outcome) and general anxiety (exploratory analysis) over time were examined using linear mixed-effects models (LMMs). The LMMs included all available observations from the 16 participants. Measurement time point was included as a categorical fixed effect, and a participant-specific random intercept was included to account for the dependency of repeated observations within participants. Given the small sample size and the four categorical measurement occasions, random slopes were not included. Models were estimated using restricted maximum likelihood estimation (REML). Degrees of freedom were estimated using the Satterthwaite approximation. Alternative model specifications were examined for both outcomes, including a diagonal repeated-measures covariance structure, compound symmetry, a participant-specific random-intercept model, and an unstructured covariance structure. Model fit was compared using information criteria. For both outcomes, the random-intercept and compound-symmetry specifications yielded identical fit indices and substantially better fit than the diagonal covariance structure, while the unstructured model was more highly parameterized and showed less favorable information criteria. The participant-specific random-intercept model was therefore retained as the more parsimonious specification.

Model assumptions were evaluated by visual inspection of residual histograms, Q–Q plots, and plots of residuals against predicted values. Standardized residuals were inspected for extreme observations. Pairwise comparisons between assessment occasions were conducted using estimated marginal means with Bonferroni adjustment for multiple comparisons. Effect sizes for changes over time were calculated as repeated measures (Cohen’s *d*_*RM*_), accounting for the dependency between repeated observations [32]. Mean differences between assessment occasions were standardized using the standard deviation of the earlier assessment and corrected for the correlation between repeated measurements according to $\:{d}_{RM}=\left({M}_{1}-{M}_{2}\right)/\left[S{D}_{1}\sqrt{2\left(1-r\right)}\right]$, where * r* denotes the Pearson correlation between the two assessment occasions. Effect sizes and corresponding 95% confidence intervals were calculated using the Psychometrica online effect-size calculator. Due to the small sample size, we chose not to include the exact time intervals between data collection in the model. Changes in diagnostic status over time were analyzed using Cochran’s Q test for related binary outcomes. Pairwise changes between assessment points were examined using exact McNemar tests. Given the pilot nature of the study and the small sample size, no adjustment for multiple comparisons was applied. Exploratory associations between dimensional treatment outcome (defined as the changes in SM symptom severity from first baseline to follow-up) and demographic as well as clinical baseline variables (age, retrospective behavioral inhibition, symptom severity at baseline, and child sex) were analyzed using Spearman rank correlations. The 15 children who participated in all assessments were included in the analyses of diagnostic status and exploratory correlations. Statistical tests were two-tailed, and statistical significance was set at *p* <.05. All analyses were conducted in IBM SPSS Statistics 29.0.2.0 (IBM Corp., Armonk, NY, USA).

## Results

Means and standard deviations of continuous dependent variables and diagnostic status of the dichotomous outcome are demonstrated in Table 3.

**Table 3:** Descriptive statistics across assessment points

	Baseline 1	Baseline 2	Post	Follow-up
Mean FSSM severity scale	2.21 (0.51)	1.97 (0.48)	1.61 (0.54)	1.44 (0.60)
BAV total T-score	63.38 (11.66)	61.25 (13.38)	58.00 (15.22)	51.13 (13.11)
No SM according to clinical interview (%)	0	13	27	47

*FSSM* Frankfurt Scale for the Assessment of Selective Mutism, *BAV *The Bochum Anxiety Assessment for Preschool and Elementary School-Aged Children

### Primary outcome: SM severity

An LMM revealed a significant effect of time on SM symptom severity (*F*(3, 43.16) = 12.76, *p* <.001). Pairwise comparisons indicated no significant change between the two baseline assessments (*p* =.070), although the observed reduction was of moderate magnitude (*d*_*RM*_ = 0.52, 95% CI [−0.11, 1.30]). Significant reductions in SM symptom severity emerged from baseline 1 to post-treatment (*p* <.001, *d*_*RM*_ = 1.03, 95% CI [0.14, 1.62]) and follow-up (*p* <.001, *d*_*RM*_ = 1.69, 95% CI [0.92, 2.53]). Symptom reduction from baseline 2 to post-treatment (*p* =.016, *d*_*RM*_ = 1.03, 95% CI [0.59, 2.11]) was also significant. From baseline 2 to follow-up (*p* <.001, *d*_*RM*_ = 0.98, 95% CI [0.01, 1.53]), SM symptom severity was significantly reduced. No significant difference was found between post-treatment and follow-up (*p* =.192, *d*_*RM*_ = 0.36, 95% CI [−0.36, 1.09]).

### Secondary outcomes: remission rate

**Fig. 3:**
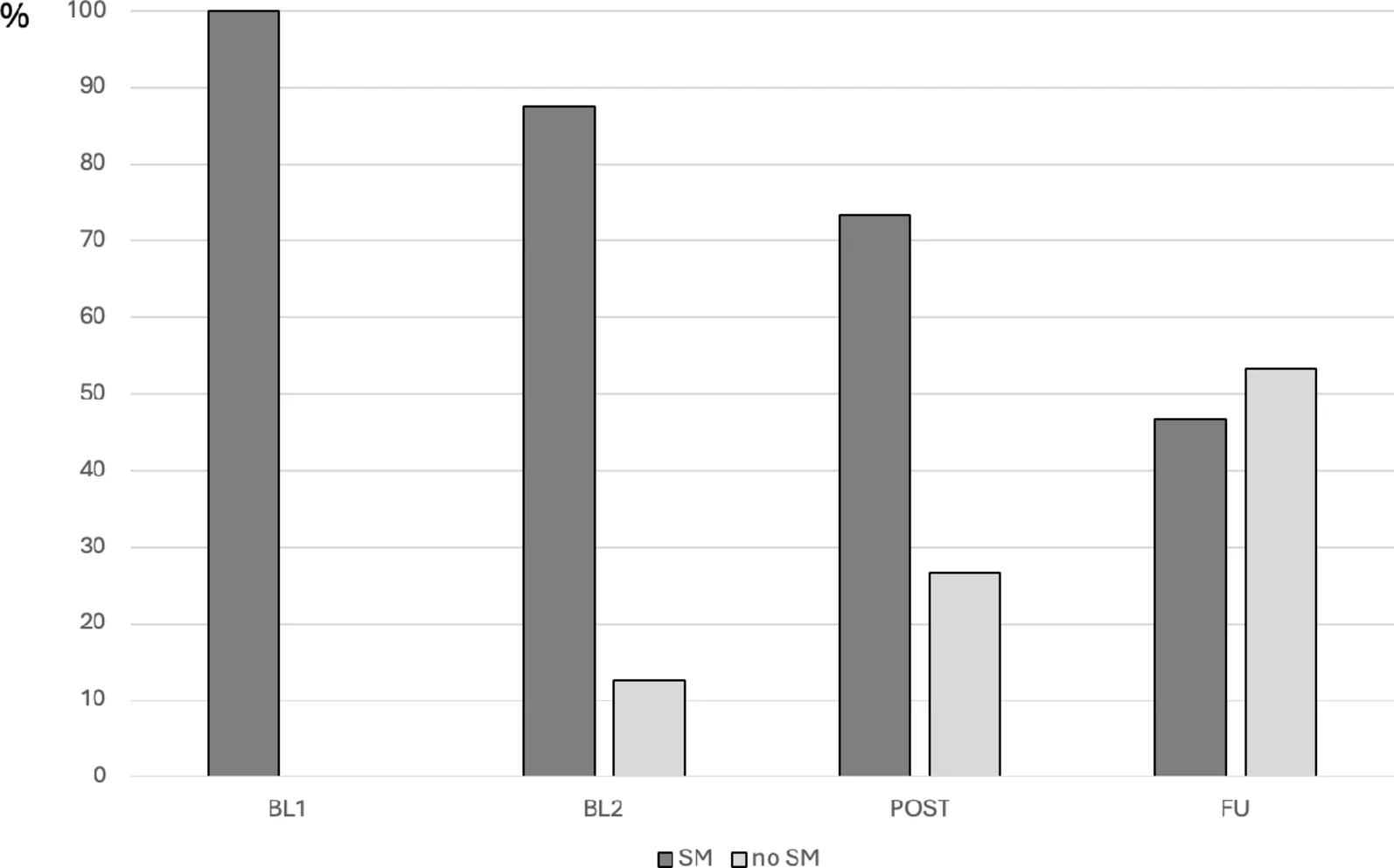
Diagnostic status in percent at various assessment points according to clinical interview. BL1 = Baseline 1, BL2 = Baseline 2, FU = Follow-up, SM = selective mutism

Remission rates increased over time with 0% of children without SM at first baseline to 47% at follow-up. A Cochran’s Q test revealed a significant overall change in diagnostic status across assessments (Q(3) = 11.07, *p* =.011). Pairwise McNemar tests only reached statistical significance for the comparison between first baseline and follow-up (*p* =.016). Figure 3 shows the proportion of children who met the diagnostic criteria for SM in the clinical interview across the various assessment points.

### Exploratory analyses: general anxiety and correlations

An LMM revealed a significant effect of time on general anxiety (*F*(3, 43.16) = 2.72, *p* <.001). Pairwise comparisons indicated no significant change between the two baseline assessments (*p* = 1.000) and between baseline 2 and post-treatment (*p* =.486). General anxiety was significantly lower at post-treatment than at baseline 1 (*p* =.040). At follow-up, general anxiety was significantly lower than at both baseline 1 (*p* <.001) and baseline 2 (*p* <.001), and was also significantly lower than at post-treatment (*p* =.009). Exploratory correlation analyses did not reveal significant associations between dimensional treatment outcome and age (*r*_*s*_ = 0.304), retrospective behavioral inhibition (*r*_*s*_ = − 0.020), symptom severity at baseline (*r*_*s*_ = 0.368), and child sex (*r*_*s*_ = − 0.283; with females coded as 1 and males as 2), all *p* >.05.

## Discussion

The current study was designed to assess changes in SM outcomes following intensive group intervention for children with SM. By examining outcomes within a German healthcare context, the study extends previous findings from North America and provides preliminary evidence regarding their generalizability across healthcare systems, reporting clinically meaningful outcomes.

Firstly, we expected SM symptom severity to decrease over time with significant differences between baseline and post-assessment as well as baseline and follow-up assessment. Our findings indeed indicate such improvements with large effect sizes (*d*_*RM*_ = 1.03 and *d*_*RM*_ = 1.69, respectively) and therefore are in line with two other studies conducted in North America [19, 20, 33]. This finding suggests that intensive group interventions based on PCIT-SM principles can be implemented in the German healthcare system. From a theoretical perspective, this intervention format may be particularly suitable because it combines measures that aim at a gradual reduction of behavioral freezing with repeated exposure to speech-demanding situations in a predictable and supportive social environment. Therefore, PCIT-SM may target processes that have been proposed to contribute to silence in SM [7, 8]. Divergent findings of one other study may partly relate to either a very small sample size (*N* = 9) or the adoption of a remote intervention format [21]. The magnitude of symptom reduction observed in the present study was comparable to that reported by Kupferberg et al. [20] and [18] and exceeded those reported in other studies assessing treatment effects in SM. One possible explanation is the use of a more comprehensive symptom measure that included a severity scale and a larger number of items, potentially allowing for a more sensitive assessment of change. However, comparisons across studies should be interpreted cautiously given the small sample sizes.

Secondly, we had expected to find a significant remission rate from baseline to follow-up assessment. Findings revealed an overall change in diagnostic status across assessments with a significant difference between first baseline and follow-up assessment and a 47% remission rate, supporting our hypothesis. This almost perfectly aligns with the findings from [19] who found a remission rate of 46% at follow-up assessment in their study with no significant change at post-assessment. The other studies did not report effects on diagnostic status. The delayed improvement in diagnostic status may be explained by the structure of the intervention. During the intensive treatment week, parents were only partially involved in treatment sessions and therefore had limited opportunities to observe changes in their children’s speech behavior. As a result, symptom improvements may not have been sufficiently evident immediately after treatment to justify a change in diagnostic status. Furthermore, the skills taught in the parent coaching may have helped implement changes in daily life and contributed to a more significant reduction in symptoms.

Thirdly, we assessed general anxiety changes over time and correlations with treatment outcome in an exploratory manner. The omnibus test for general anxiety over time revealed significant changes. General anxiety was significantly lower at post-treatment than at baseline 1 and was significantly lower at follow-up than at baseline 1, baseline 2, and post-treatment. No statistically significant difference was observed between baseline 1 and baseline 2 or between baseline 2 and post-treatment. These findings suggest that reductions in general anxiety may have emerged more gradually than changes in SM symptom severity. This result partly aligns with the findings from [19] who found an improvement in social anxiety at post-assessment with a medium effect size. The pattern of findings suggests that changes in speech-related symptoms may precede changes in general anxiety. None of the sociodemographic and other variables correlated with the treatment outcome, which may be due to the small sample size and the resulting lack of statistical power. In particular, age was not significantly associated with treatment outcome. However, given the small sample size and the restricted age range, this finding does not allow conclusions regarding the appropriateness of the selected age range. Future adequately powered studies should investigate whether age or developmental stage moderates treatment response.

Importantly, the intervention could be implemented in a German clinical context without major adaptations to its core therapeutic components. The magnitude of symptom reduction and the remission rate observed in our sample were broadly comparable to those reported in previous studies from the United States [18, 19]. This provides preliminary support for the applicability of PCIT-SM-based intensive treatment in a different healthcare context in which it was originally developed.

### Limitations

Our study has several limitations to consider. First, the sample size was small, which did not allow for inclusion of other variables such as the interindividual time differences in assessments and might have led to a lack of power to assess the effect of possible moderators or mediators on treatment outcomes. Small sample size limits the precision of the effect estimates, as reflected in the relatively wide confidence intervals, and may have reduced the ability to detect smaller changes between individual assessment occasions. Accordingly, non-significant findings should not be interpreted as evidence of the absence of an effect. Future studies should therefore evaluate the intervention in larger, adequately powered samples and, ideally, use controlled study designs. Furthermore, generalizability is limited due to the small sample size. Secondly, we did not have a control group but used a repeated-measures design with two baseline assessments instead. Although SM symptomatology is quite stable over the time course of nine months in early childhood [2, 15, 16], it cannot fully be ruled out that spontaneous remissions would have happened without any intervention with this design. Indeed, we observed some symptom reduction already between the two baseline assessments. Several explanations for this reduction are conceivable, including natural symptom fluctuation, regression to the mean, and measurement variability associated with repeated assessment. Increased motivation or engagement associated with participation in the study may also have contributed. A sufficiently powered controlled trial is needed to better determine the extent to which such changes can be attributed to different causes. Moreover, a design with independent clinical groups would have allowed us to conduct an analytic evaluation of different aspects of treatment. This would have provided a basis for drawing conclusions regarding the significance and effective components of the intervention, as well as, indirectly, the underlying mechanisms. Another limitation is the selection of participants from the sample screened. Apart from families who withdrew their participation on their own initiative, this selection was based primarily on the goal of forming a group of participants of similar ages, rather than on a random selection. Our primary outcome SM symptom severity was a parent-rated questionnaire which may not fully capture speaking behavior across different social contexts. Here, an objective behavioral observation of child behavior in different settings would have been an optimal addition. Lastly, the follow-up period was very short with only three months between termination of the treatment and follow-up assessment. A larger follow-up period would have allowed for estimating the stability of treatment effects.

### Conclusions

Overall, the present study provides preliminary evidence that intensive group interventions based on PCIT-SM principles may represent a promising treatment approach for children with SM in a German clinical sample. Future research should extend the current findings in larger controlled trials and investigate the hypothesized roles of behavioral freeze regulation and avoidance reduction more directly to further refine treatment approaches for SM. Furthermore, future studies should investigate whether intensive group interventions are comparably effective to individual treatments or should be employed in addition to individual treatments as enhancers of treatment success.

## Data Availability

The datasets generated during the current study are not publicly available because they contain potentially identifiable clinical information but are available from the corresponding author on reasonable request.
